# Ribonucleotide reductase M1 regulates the cell cycle via minichromosome maintenance proteins

**DOI:** 10.1016/j.gendis.2025.101927

**Published:** 2025-11-08

**Authors:** Agnes Malysa, Jenny M. Kreahling, Scott N. Freeman, Bin Bao, Jiawei Zhao, Xiaohong Zhang, Manohar Ratnam, Gerold Bepler

**Affiliations:** a Karmanos Cancer Institute and Department of Oncology, Wayne State University, Detroit, MI 48201, USA; b Department of Thoracic Oncology, H. Lee Moffitt Cancer Center and Research Institute, Tampa, FL 33612, USA

Ribonucleotide reductase (RNR) is the key enzyme converting ribonucleotides to deoxyribonucleotides.^1^ It forms a heterotetramer consisting of two regulatory subunits (R1) (encoded by RRM1) and two catalytic subunits (R2) (encoded by RRM2).^1^ Here we report that the catalytic minichromosome maintenance (MCM4/6/7) proteins,^2^ which are essential for DNA replication, interact with R1, but not R2. These studies were initiated after we had found a positive correlation between R1 expression and survival in patients with lung cancer.^3^ We found that R1 interacted with catalytic MCM4/6/7 in M-phase cells and MCM7 in non-M-phase cells, and that R2 overexpression did not disrupt these R1/MCM interactions. While knockdown of R1 decreased the protein and mRNA levels of MCM7 and arrested cells at the G2/M phase, overexpression of MCM7 in R1 knockdown cells relieved the G2/M arrest, leading to cell proliferation. dNTP pools do not affect cell growth in control and R1 knockdown cells, suggesting that R1's function in RNR does not influence R1's function in cell proliferation. Furthermore, there was a positive correlation between R1 and MCM7 expression in a cohort of lung cancer patient samples. Overall, our findings suggest that R1 regulates cell cycle progression through modulation of MCM7 levels, implicating that RNR and MCMs collaborate in cell cycle regulation.

While investigating putative RRM1-interacting proteins by mass spectrometry and yeast-two hybrid screens, we identified sequences corresponding to MCM6 and MCM7 that co-precipitated in various cancer and non-cancer cell lines. We immunoprecipitated endogenous R1 from human lung (H23, A549) cancer cell lines and the human embryonic kidney-derived cell line HEK293 and immunoblotted for MCM2-7 to confirm this finding and to determine if additional MCM proteins co-precipitate with R1. MCM4/6/7, but not MCM2/3/5, co-precipitated with R1 (Fig. 1A). Using the same technology, we found no evidence for an interaction between R2 or p53R2, the catalytic subunits of ribonucleotide reductase, and these six MCMs. We also performed a reverse immunoprecipitation and found that each catalytic MCM (4/6/7) pulled down R1 (Fig. S1A).

**Figure 1:**
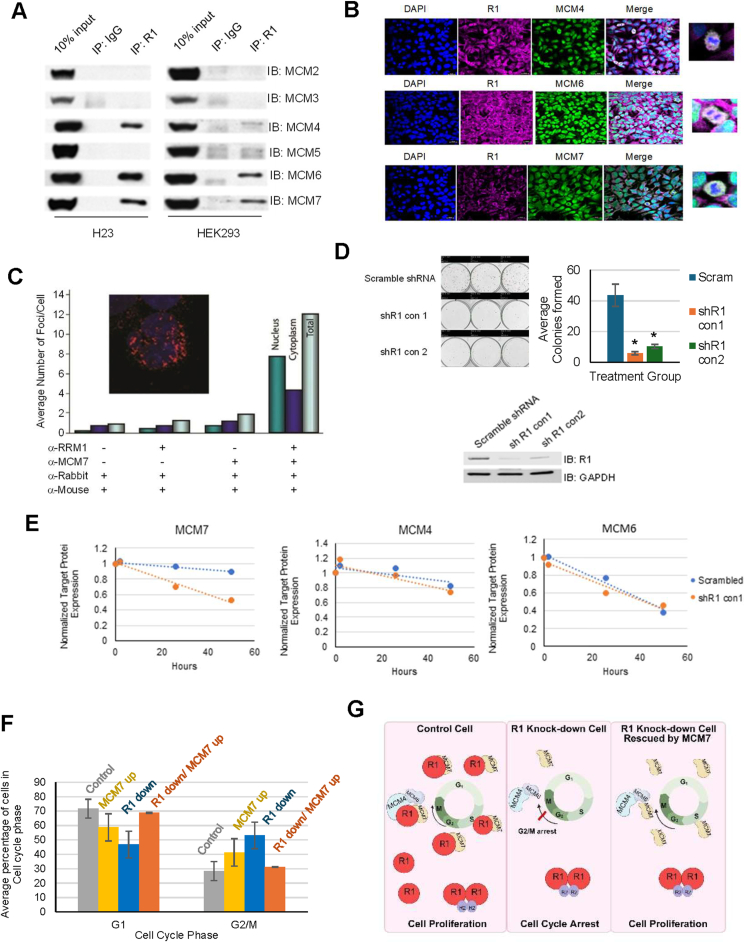
RRM1 regulates cell proliferation by regulating MCM7 levels. **(A)** RRM1 binds to MCM4, MCM6, and MCM7. **(A)** shows the immunoblots of whole cell lysates (Input) and immunoprecipitates with antibodies to RRM1 or control IgG, using the anti-MCM2, anti-MCM3, anti-MCM4, anti-MCM5, anti-MCM6, and anti-MCM7 antibodies as indicated from H23 and HEK293 cell lines. **(B)** RRM1 colocalizes with MCM4, MCM6, and MCM7 in mitotic HEK293 cells, while RRM1 also colocalizes with MCM7 in the cytoplasm in non-mitotic HEK293 cells. RRM1 colocalizes with MCM4 (upper panel), MCM6 (middle panel), and MCM7 (lower panel) in mitotic HEK293 cells. RRM1 also colocalizes with MCM7 (lower panel) in the cytoplasm in non-mitotic HEK293 cells. Representative immunofluorescence images were taken at 40 × oil magnification. For a higher quality image, please see Figure S8. **(C)** Intracellular interaction of RRM1 and MCM7 by *in situ* proximity ligation assay in cell line H23. Bars indicate the average number of foci per cell and cellular compartment. Using no primary antibody or only primary antibody to RRM1 or MCM7 resulted in an average number of two or fewer background foci per cell. Using primary antibodies to RRM1 and MCM7 resulted in an average of 12 foci per cell. Foci were observed in the nuclear and cytoplasmic compartments. The insert shows an image of a cell with predominantly nuclear foci. **(D)** Knockdown of R1 reduces cell growth in H23 cells. R1 knockdown in H23 with 1 mM IPTG and growth medium supplementation with 50 mM dNTP inhibits cell proliferation in colony formation assays (upper left panel), with the bar chart depicting trends (upper right panel). The Western blot shows the efficiency of R1 knockdown by two shRNAs (lower panel). The asterisk indicates *P* < 0.05 by Student's *t*-test. **(E)** Knockdown of R1 reduces the protein level of MCM7 but not MCM4 and MCM6. A549 shR1 cells were induced with 1 mM IPTG and supplemented with 50 mM dNTP. Zero-, 26-, and 50-h timepoints were collected for MCM protein expression. GAPDH-normalized levels for MCM7, MCM4, and MCM6 are depicted, showing that only MCM7 levels decline faster in cells with reduced R1 levels (Western blots in Fig. S9). **(F)** MCM7 overexpression rescues G2/M arrest in R1 knockdown HEK293 cells. Flow cytometry analysis showed MCM7 overexpression (MCM7 up), R1 knockdown, and MCM7 overexpression in R1 knockdown HEK293 cells. MCM7 overexpression rescued G2/M arrest in cells after 72 h of R1 knockdown in HEK293 cells. HEK293 shR1 cells were treated with (+) and without (−) IPTG in the presence of MCM7 overexpression (+) and control vector (−). **(G)** A working model of how R1, by forming two complexes, R1/MCM4/MCM6/MCM7 and R1/MCM7, regulates the cell cycle and cell proliferation (created by BioRender).

Since co-precipitation does not demonstrate direct protein–protein interaction, we utilized an *in vitro* binding assay to determine which of the three MCMs (4/6/7) directly associates with R1. Biotin-histidine(His)-tagged R1 produced from baculovirus-infected insect cells was purified, bound to streptavidin beads, and utilized in a binding assay with hemagglutinin(HA)-tagged MCM4, MCM6, or MCM7 protein prepared using an *in vitro* transcription/translation reaction. All three MCMs were efficiently produced (Fig. S1B). We found MCM4 and MCM7 to interact directly with R1 but observed no evidence for a direct interaction between MCM6 and R1 (Fig. S1C). These results indicate that the MCM4/6/7 complex interacts with RRM1 through direct binding of MCM4 and MCM7.

We used confocal microscopy to assess cellular localization of the complex in HEK293 cells and observed that while R1 colocalized with MCM4, MCM6, and MCM7 only in dividing (M-phase) cells (Fig. 1B), R1 colocalized only with MCM7 predominantly in the cytoplasm in non-M phase cells (Fig. 1B). These observations led us to conclude that R1 formed two distinct complexes in cells, R1/MCM7 in non-dividing cells, and R1/MCM4/6/7 during mitosis. We deployed an *in situ* proximity ligation assay to visualize and quantify the intracellular interaction between endogenous RRM1 and MCM7. By this method, immunofluorescent foci are observed only when two different target-specific antibodies are less than 40 nm apart, thus indicating a protein–protein interaction. After confirming that the RRM1 and MCM7 antibodies individually provided a robust and specific signal, we proceeded with the co-localization investigation. We observed on average 12 foci per cell, a 6-fold increase over the background, with foci located in the cytoplasm and the nucleus (Fig. 1C). These results confirm an interaction between RRM1 and MCM7 and demonstrate that it occurs in the cytoplasm as well as in the nucleus, an observation that our lab has witnessed.^3^

Since RNR and the MCMs function primarily during S-phase, we surmised that the R1/MCM complexes would dissociate with increased availability of R2, since R2 expression peaks during S-phase. To investigate this assumption, we generated doxycycline-inducible R2 overexpressing cells (OER2) and immunoprecipitated these cells and control cells (ORF) with R1. In contrast to our expectation, the R1/MCM complexes remained intact, and it appeared that there was an increase in R1 complexes (Fig. S2A). This may be explained by an increase in the synthesis of R1 during the S-phase. To further investigate if increased R1 leads to enhanced R1/MCM7 complex formation, we immunoprecipitated R1 overexpressing (OER1) cells with MCM7 and observed no difference in the level of complexes (Fig. S2B), meaning that R1 complex formation in these cells is limited by MCM7 expression and not R1 expression.

To understand the potential function of these R1 complexes, we performed proliferation assays in inducible R1 knockdown cells and supplemented these stable cells with dNTPs to circumvent effects caused by defective RNR function. We performed MTT and colony formation assays and found a statistically significant decrease in cell growth (*P* < 0.001) (Fig. S3A and S3B) and colony formation (Fig. 1D). To ensure that our dNTP supplementation was adequate, we assessed the concentrations of dNTPs in control and knockdown cells and observed no differences (Fig. S4).

To determine whether the level of R1 expression in our experimental cells was comparable to that seen in clinical specimens, we created a cell line microarray paraffin block with formalin-fixed wild-type H23 and A549 cells. Sections of this block were stained with R1 (H-scores were determined) and compared to those observed in a lung cancer tissue microarray.^3^ The H-scores for R1 expression in both cell lines were 150, and those in the clinical specimens ranged from 0 to 163, indicating that R1 levels in our experimental system are at the high end of those found clinically in lung cancer, yet clinically relevant.

We studied if R1 influenced MCM7 protein levels in R1 knockdown and control A549 cells with dNTP supplementation and observed that control cells showed stable R1 and MCM7 levels; however, upon R1 knockdown, MCM7 protein and mRNA levels began to decline as soon as R1 levels declined (Fig. 1E; Fig. S5). Moreover, we observed a positive correlation between R1 and MCM7/4/6 in a cohort of lung cancer patient samples (Fig. S6), supporting the possibility that R1 regulates MCM7 (and possibly MCM4 and MCM6). To assess whether MCM7 overexpression could rescue the growth defects caused by R1 knockdown, we used flow-cytometric cell cycle analysis. While MCM7 overexpression alone had no significant impact on cell cycle distribution, it reversed the G2/M arrest seen in R1 knockdown cells to the levels observed in control cells (Fig. 1F; Fig. S7).

Our results, demonstrating a direct interaction between R1 and the catalytic MCMs, provide a novel link between DNA replication and deoxynucleotide synthesis and suggest that R1 may not only regulate ribonucleotide reductase function but also cell cycle progression via the catalytic MCMs through the formation of two distinct complexes; one with MCM7, an MCM that is associated with cell cycle regulation,^4^ and the other with MCM4/6/7. The former leads to regulation of MCM7 mRNA and protein levels, and the latter with MCM4/6/7 possibly securing M-phase progression (Fig. 1G). Our studies have laid a solid foundation for further exploration of detailed mechanisms on how R1 and MCMs simultaneously modulate cell cycle progression, genome maintenance, and DNA repair, which will help in achieving the goal of finding ways to prevent and treat diseases characterized by abnormal cell growth and genome damage.

## CRediT authorship contribution statement

**Agnes Malysa:** Writing – review & editing, Writing – original draft, Visualization, Validation, Methodology, Investigation, Formal analysis, Data curation, Conceptualization. **Jenny M. Kreahling:** Methodology, Investigation, Data curation. **Scott N. Freeman:** Methodology, Investigation, Data curation. **Bin Bao:** Methodology, Data curation. **Jiawei Zhao:** Investigation. **Xiaohong Zhang:** Writing – review & editing, Supervision. **Manohar Ratnam:** Supervision, Conceptualization. **Gerold Bepler:** Writing – review & editing, Writing – original draft, Supervision, Project administration, Conceptualization.

## Funding

This research was supported by the US NIH research grants P30-CA022453, R01-CA102726, R01-CA129343, and R50-CA251068-01.

## Conflict of interests

Xiaohong (Mary) Zhang is an associate editor of *Genes & Diseases* and was not involved in the editorial review or the decision to publish this article. All authors declare that there are no competing interests.
