# Supplementary material for: Ribonucleotide reductase M1 regulates the cell cycle via minichromosome maintenance proteins
Source: Genes Dis. 2025 Nov 8;13(4):101927. doi: 10.1016/j.gendis.2025.101927 (PMC12995475; doi:10.1016/j.gendis.2025.101927)
Supplement: Multimedia component 1 [file mmc1.docx]

**Supplemental Materials**

**MATHERIALS AND METHODS**

**Cell lines**

The cell lines (H23, A549, MCF7, HEK293) and human foreskin fibroblasts (HFF) were obtained from ATCC, and their authenticity was confirmed by DNA fingerprint analysis. They were cultured in growth medium (RPMI-1640 for H23, A549, and MCF and MEM for HEK293) supplemented with 10% FBS and 1% Penicillin-Streptomycin.

**Knock-down and overexpression cells**

Knock-down and overexpressing tetracycline-inducible vectors for R1 and R2 were created and packaged into lentivirus by Vector Builder (<http://en.vectorbuilder.com>). H23, A549, and HEK293 wildtype cells were infected and selected for shR1 and shR2 knockdown constructs with 1 mg/ml puromycin for two weeks. Knockdown was induced with 1 mM IPTG (Thermo Fisher, R1171) in the presence of 50 μM dNTPs^1^ for 3-4 days. For overexpression, HEK293 were first transduced with the transactivator plasmid followed by hygromycin selection for two weeks and then the R1, R2, or control inducible (ORF) plasmids followed by two weeks of 1 mg/ml puromycin and 100 mg/ml hygromycin selection. Overexpression was induced with 250 ng/ml doxycycline for 2 days. Western blots were used to validate the induction of knockdown and overexpression in all cell lines.

**Immunoprecipitation**

Cells were immunoprecipitated with anti-R1 (Antibodies.com, a13328 and Thermo Fisher, UM800059CF), anti-R2 (Antibodies.com, a13328), anti-MCM4 (Fortis Life Sciences, A300-125A), anti-MCM6 (Fortis Life Sciences, A300-127A), and anti-MCM7 (Foritis Life Sciences, A300-128A) using the Pierce Classic Magnetic IP/Co-IP Kit (Thermo Fisher, 88804).

**Densitometry**

Densitometry was performed to measure GAPDH-normalized protein expression according to <http://www.navbo.info/DensitometricAnalysys-NIHimage.pdf>.

***In Vitro* binding assay**

For *in vitro* binding assays, biotin-histidine (His)-tagged R1 protein was expressed in baculovirus, purified, and bound to Dynabeads® M-280 streptavidin beads (Invitrogen, 112.05D). Hemagglutinin (HA)-tagged MCM proteins were synthesized using coupled in vitro transcription and translation in rabbit reticulocyte lysates (Promega, L4610). Lysates were incubated with biotin-His-R1 bound streptavidin beads overnight at 4˚C in NETN buffer, and complexes were resolved on denaturing polyacrylamide gels. Antibodies utilized for immunoblotting included those specific for the His-tag (Santa Cruz, sc-803) and HA-tag (Covance, MMS-101R).

**Immunofluorescence**

Cells were seeded on poly-D-lysine coated (50 μg/ml, Thermo Fisher, A3890401) slides in 6-well plates, washed with PBS, fixed/permeabilized with ice cold methanol, blocked with 3% BSA+PBS, and incubated overnight at 4^o^C in a hydration chamber with the following antibodies: anti-R1 (Thermo Fisher, # UM800059CF), anti-R2 (Thermo Fisher, PA5-92365), anti-MCM4 (Thermo Fisher, # PA5-29039), anti-MCM6 (Thermo Fisher, 13347-2-AP), anti-MCM7 (Thermo Fisher, 11225-1-AP), and anti-Ki67 (Cell Signaling 9129, clone D3B5). The next day, slides were washed with PBS followed by secondary antibody incubation (Alexa Fluor 488; Thermo Fisher, A-21206 and Alexa Fluor 647; Thermo Fisher, A-31571) for 1 hr at room temperature, followed by another 3 cycles of PBS washes. Slides were mounted with Prolong Gold Antifade with DAPI (Thermo Fisher, P36931). Images were acquired using a Zeiss LSM 780 microscope with 40x oil magnification.

**Proximity ligation assay**

The proximity ligation assay (PLA) was conducted using the Duolink™ *in situ* PLA kit 563 (Olink Biosciences) following the manufacturer’s protocol with the following alterations: fixation with 4% paraformaldehyde, permeabilization with 0.5% Triton-X, and blocking and antibody dilution with 2% BSA in PBS. The primary antibodies used in this assay were specific for RRM1 (a proprietary rabbit monoclonal antibody, C6-2, generated using an N-terminal peptide sequence, and a commercial goat antiserum, sc-11733, from Santa Cruz Biotechnology) and MCM7 (a commercial mouse monoclonal antibody, Santa Cruz, sc-46687). Two microscopic fields consisting of 10 z-sections each were obtained with a 25x oil immersion objective for every sample, and images were analyzed using BlobFinder software (<http://www.cb.uu.se/~amin/BlobFinder/>, Uppsala, Sweden), and the average number of foci per cell was calculated.

**MTT assay**

For shR1 and shR2 knock-down cell lines, 1,000-2,000 cells (scrambled and shR1) were seeded into 96-well plates and allowed to attach for 24 hr. Next day, cells were treated with 1 mM IPTG and 50 μM dNTP. We also simultaneously seeded 25,000-50,000 cells (scrambled and shR1) into 6-well plates to evaluate protein expression by Western blot. On Days 1, 2, or 3 following induction, MTT (3-(4, 5-dimethylthiazol-2-yl)-2, 5-diphenyltetrazolium bromide) was added (Thermo Fisher, # M6494), plates were incubated for 2 hr at 37^o^C, then media was removed and 200 mL DMSO was added. The plates were agitated for 15 min, and the optical density at 570 nM was read. At each timepoint, the cells seeded for Western blot analysis were lysed using RIPA buffer (Sigma, # R0278) with 1x Protease inhibitor (Thermo Fisher, PI87786) and subjected to protein quantification (using the BCA assay; Thermo Fisher, 23250). Equal amounts of protein were run for Western blots and bands were visualized by the ChemiDoc (Biorad).

**Colony formation assay**

All cell lines were seeded at a density of 1,000 cells per well using Nunclon Delta -coated 6-well plates (Thermo Fisher, # 140675) and allowed to attach for 24 hr. Knock-down R1 and control (scrambled) cells were treated with and without 1 mM IPTG supplemented with 50 μM dNTP. Once 50-cell colonies appeared, cells were fixed, stained with crystal violet solution (Sigma, CG158), 25% methanol), and colonies were counted using Gel Count (Scintica).

**Measuring dNTP and NTP pools**

For targeted metabolomics, H23, A549, and HEK293 control and shR1 cells were induced with 1 mM IPTG and with or without 50 μM dNTP for 96 hr and collected with ice cold 80% methanol. Metabolite measurement was performed with the B SCIEX QTRAP 6500 LC-MS/MS system. Intracellular nucleoside triphosphates and deoxynucleoside triphosphates were determined by a validated LC-MS/MS method published previously by us^1^.

**R1 knockdown Study**

A549 knock-down R1 and control cells were seeded in 6-well plates and allowed to attach and equilibrate for 24 hr. This was followed by a 24 hr incubation with medium containing 1 mM IPTG and 50 μM dNTPs for knock-down induction. To assess the effects of R1 knockdown on MCM7 expression, cells were collected at 0, 2, 26 and 50 hr. Cells were lysed with RIPA (Sigma, R0278) and 1x protease inhibitor (Thermo Fisher, PI87786) and protein levels were assessed using the BCA assay (Thermo Fisher, 23250). Analysis for calculating half-life was done according to <https://bio-protocol.org/en/bpdetail?id=1919&type=0>).

**Flow-cytometry analysis**

HEK234 shR1 and control cells were seeded and allowed to attach for 24 hr. Control and treatment cells were induced for R1 knock-down (1mM IPTG and 50 μM NTPs) and infected with pCMV empty vector and pCMV-MCM7 overexpression vector (Vector Builder, MOI: 40) at the same time and incubated for 72 hr and 92 hr followed by preparation for Western blot analysis as described above. Flow samples were washed with PBS followed by fixation with ice-cold 70% ethanol, and samples were stored in -20^o^C. On the day of flow analyses, samples were washed twice with PBS and stained with Propidium Iodide/RNAse (Ready-to-Use solution) supplemented with 1% Triton-X (BD Pharmigen, 550825). Flow samples underwent cell cycle analysis.

**Patient samples and Immunohistochemistry**

All human subject studies were under the approval of Wayne State University (WSU) Institutional Review Boards (IRBs).  Our tissue micro array^2^ that consists of 252 NSCLC patients was stained for RRM1 (1:100, overnight 4°C, Antibodies.com, Catalog: a13328), MCM4 (1:100, overnight 4°C, C-10, Santa Cruz, Catalog#: sc-48407), MCM6 (1:100, overnight 4°C, H8, Santa Cruz, Catalog#: sc-393618 ), and MCM7 (1:100, overnight 4°C, clone 141.2, Santa Cruz, Catalog#: sc-9966), where expression was visualized by DAB substrate (*Agilent,* Catalog#: K3468) and scored by 3D Histech (<https://www.3dhistech.com/>). Slides were scored at the Karmanos Cancer Institute BioBanking and Correlative Sciences Shared Resource.

**Real-time PCR**

RNAs were isolated using RNeasy Mini kit (Qiagen), following the manufacturer’s instructions. The reverse transcription reaction (1,000 ng RNA in 20 μL of reaction volume for each sample) was done using a High-Capacity cDNA Reverse Transcription kit (ThermoFisher), following the manufacturer’s instructions. The PCR reaction was performed using a standard method with CybrGreen PCR solution (ThermoFisher). The delta delta Ct values were calculated for the data analysis. The primers are listed in Table S1. The 18S RNA PCR product served as an internal control.

**Statistics**

For MTT analysis, the endpoint analyzed was fold change (FC) on day 3 compared to day 1. Measurement on day 3 for each of 16 wells (obs) was divided by the average of 16 obs on day 1 in the same condition and same experiment. The mixed effect model was used to compare the FCs between treatments, namely the R1 knockdown (KD) construct #1 vs control (scrambled) and R1 KD construct#2 vs control. Multiple comparisons were adjusted with Dunnett contrasts. For all other experiments, the two-sample t-tests were performed to determine statistical significance.

| **Table S1. Primers for Real-time PCR** | |
| --- | --- |
| RRM1-Forward | 5’-GCCAGGATCGCTGTCTCTAAC-3’ |
| RRM1-Reverse | 5’-GAGAGTGTTTGCCATTATGTGGA-3’ |
| MCM7-Forward | 5’-CCTACCAGCCGATCCAGTCT-3’ |
| MCM7-Reverse | 5’-CCTCCTGAGCGGTTGGTTT-3’ |
| MCM6-Forward | 5’-TCGGGCCTTGAAAACATTCGT-3’ |
| MCM6-Reverse | 5’-TGTGTCTGGTAGGCAGGTCTT-3’ |
| 18S-Forward | 5’-GCAATTATTCCCCATGAACG-3’ |
| 18S-Reverse | 5’-GGCCTCACTAAACCATCCAA-3’ |

**A**

IP: MCM4

IP: IgG

10% Input

IP: MCM6

IP: IgG

10% Input

IP: MCM7

IP: IgG

10% Input


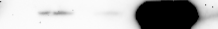

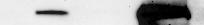

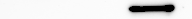

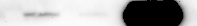

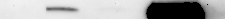


IB: MCM4 4


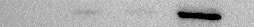

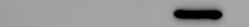

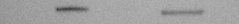

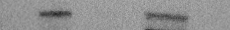

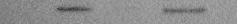


IB: R2

IB: R1

IB: MCM6 7

IB: MCM5 6


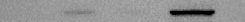

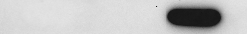

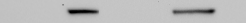

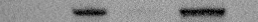

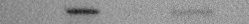


**B C**


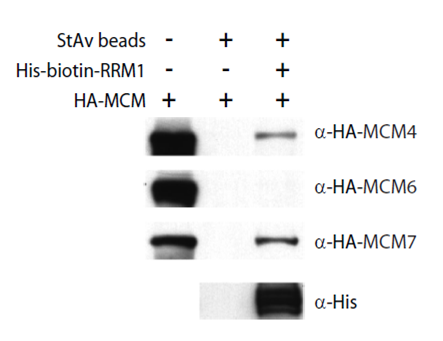


StAv beads - + +

His-biotin-RRM1 - - +

HA-MCM + + +

α-HA-MCM4

α-HA-MCM6

α-HA-MCM7

α-His


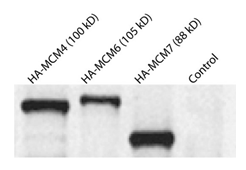


HA-MCM4 (100 kDa)

HA-MCM6 (105 kDa)

HA-MCM7 (88 kDa)

Control

**Figure S1.**  **RRM1 binds to MCM4, MCM6, and MCM7.** (A) Immunoblots of whole cell lysates (Input) and immunoprecipitates with control IgG or anti-MCM4, MCM6, and MCM7 antibodies were performed using the indicated antibodies and cell line, HEK293. (B) Immunoblot of in vitro transcribed and translated HA-tagged MCM4, 6, and 7 with anti-hemagglutinin (HA) antibody. (C) *In vitro* binding of biotin-His-tagged RRM1 and HA-tagged MCMC4, 6, and 7. Immunoblotting was done with anti-HA. Columns 1 and 2 are controls and show the presence of all three MCMs in the respective lysates (column 1) and absence in streptavidin isolates (column 2). The addition of biotin-His-tagged RRM1 to the MCM-containing lysates followed by streptavidin isolation demonstrates a direct interaction of RRM1 with MCM4 and 7, but no direct interaction with MCM6 (column 3).

**A B**

HEK293 ORF IP: MCM7

HEK293 ORF IP: IgG

HEK293 ORF 10% Input

HEK293 OER1 IP: MCM7

HEK293 OER1 IP: IgG

HEK293 OER1 10% Input

IB: R2

IB: MCM7

IB: MCM6

IB: MCM4

IB: R1


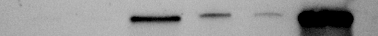

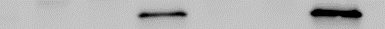

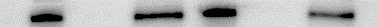

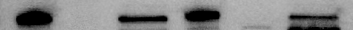

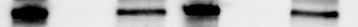


IB: R2

IB: R1

IB: MCM7

HEK293 ORF IP: R1

HEK293 ORF IP: IgG

HEK293 ORF 10% input

HEK293 OER2 IP: R1

HEK293 OER2 IP: IgG

HEK293 OER2 10% input

IB: MCM6

IB: MCM4


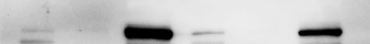

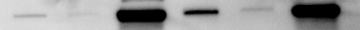

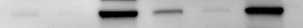

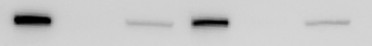

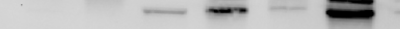


**Figure S2.**  **Overexpression of R2 does not disrupt the R1/MCM4/6/7 complex formation and Overexpression of R1 does cause an abundance of R1 complexes.** (A) R2 overexpression (OER2) does not disrupt the R1/MCM complex formation. In fact, R2 overexpression increases R1/MCM complexes (OER2) compared to control (ORF) in HEK293 cells. (B) Immunoprecipitation with MCM7 showing increased R1 in immunoprecipitates in R1 overexpressing HEK293 cells (OER1) versus control (ORF) cells. R2 and R1 overexpression was induced with 250 ng/ml doxycycline for 48 hrs.

**A**

IB: R1

IB: GAPDH

Scrambled

shR1 construct 1

shR1 construct 2

Scrambled

shR1 construct 1

shR1 construct 2

Scrambled

shR1 construct 1

shR1 construct 2

Day 1

Day 2

Day 3


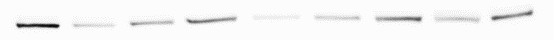

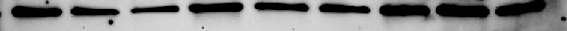


**
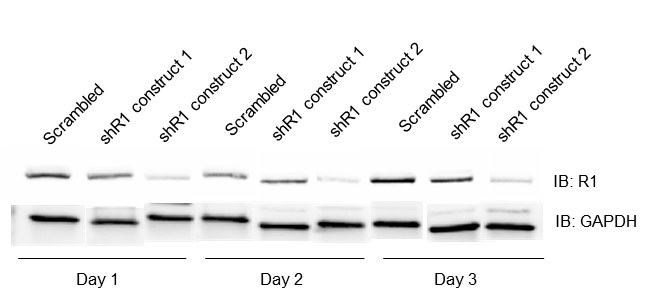
B**

**Figure S3. Knockdown of R1 reduces cell growth in HEK293 and H23 cells.** R1 knockdown in HEK293 (A) and H23 (B) with 1 mM IPTG and growth medium supplementation with 50 μM dNTP inhibits cell proliferation in MTT assays and associated Western blots.

**Figure S4. dNTP pools do not affect cell growth in control and R1 knockdown H23 cells.** R1 knockdown in H23 cells with 1 mM IPTG and growth medium supplementation with 50 μM dNTP inhibits cell proliferation in MTT assays. No difference was detected between control and R1-knockdown H23 cells in terms of cell growth by MTT assays under the condition of adding dATP, dCTP, dGTP, or dTTP.


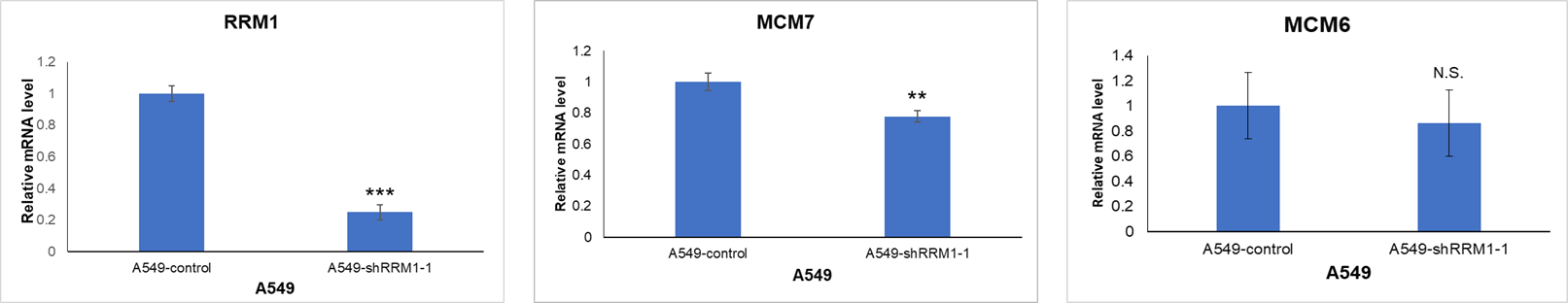


**Figure S5. R1 knockdown decreases MCM7 mRNA expression in A549 cells.**  1mM IPTG was added to the A459 cell line on day 0 and day 2 to induce RRM1 knockdown. Control and IPTG-treated cells were harvested on day 3 for real-time PCR experiments. 18S-normalized values for mRNA expression of RRM1, MCM7, and MCM6 were quantified and displayed as bar graphs. For statistical analysis, a student’s *t*-test was used. ns, *p*>0.05; *, *p*<0.05; **, *p*<0.01; ***, *p*<0.001.


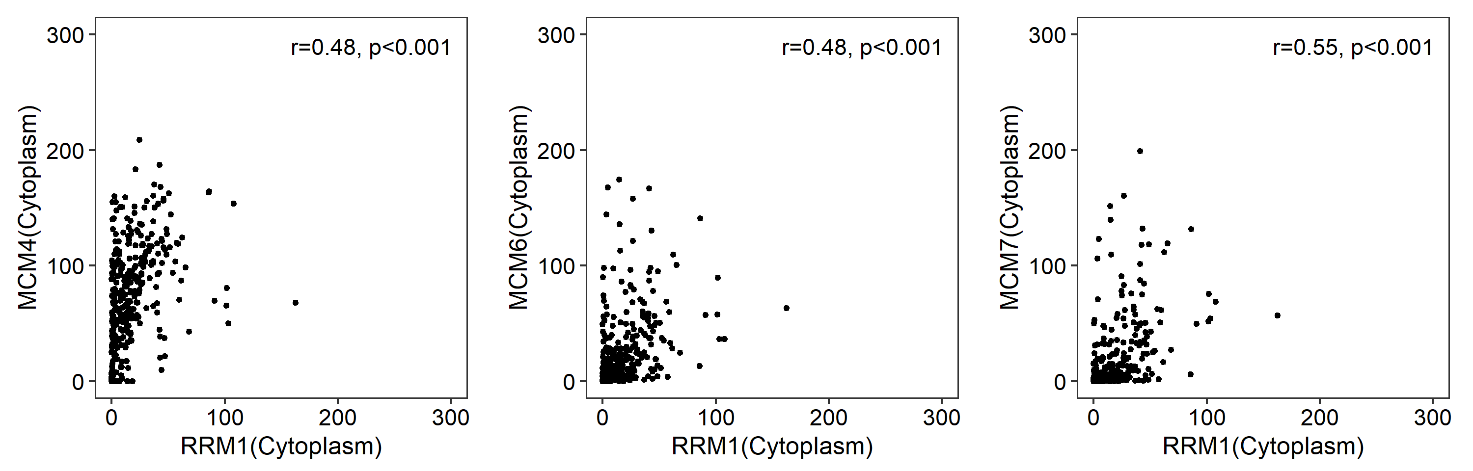
**A**


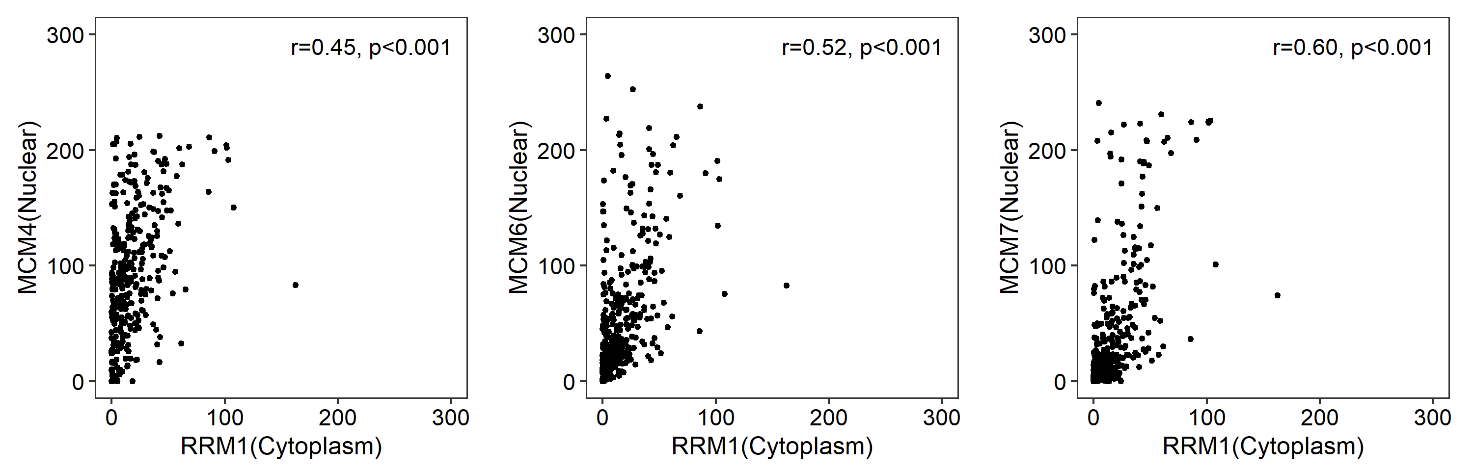
**B**

**C**


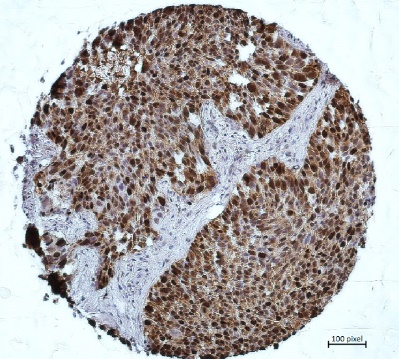

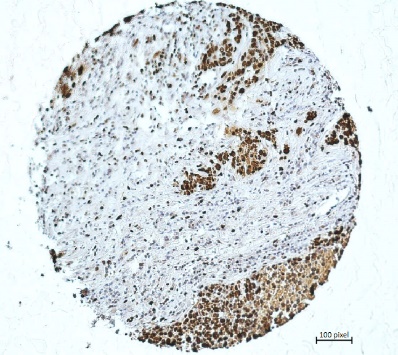

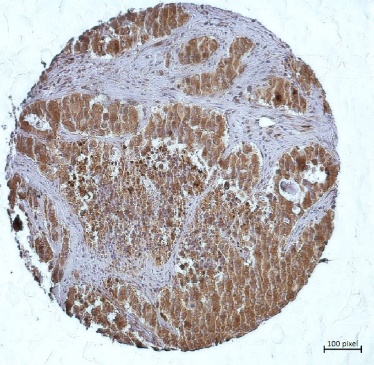


**Figure S6. There is a positive correlation between cytoplastic R1 and MCM4/6/7 in a cohort of NSCLC lung cancer patient samples.** Spearman correlation analysis shows positive correlation between (A) cytoplasm RRM1 expression with cytoplasm catalytic MCM4, MCM6, and MCM7 H scores; (B) cytoplasmic R1 and nuclear catalytic MCM4. MCM6, and MCM7 H-scores. (C), Representative 10x IHC non-small cell lung carcinoma images for MCM4, MCM6, and MCM7 (L-R).

IB: R1

IB: MCM7


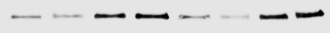

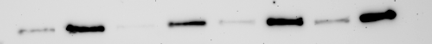

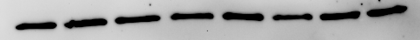

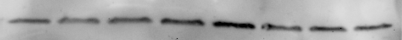


IB: MCM6


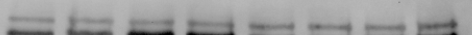


IB: MCM4


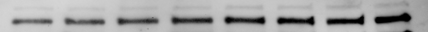


IB: GAPDH

IB: R2

MCM7 - + - +

shR1 + + - -

**Figure S7. MCM7 overexpression rescues G2/M arrest in R1 knockdown HEK293 cells.** Western blot of R1 knockdown and MCM7 overexpression in HEK293 cells. HEK293 shR1 cells were treated with (+) and without (-) IPTG in the presence of MCM7 overexpression (+) and control vector (-).

**
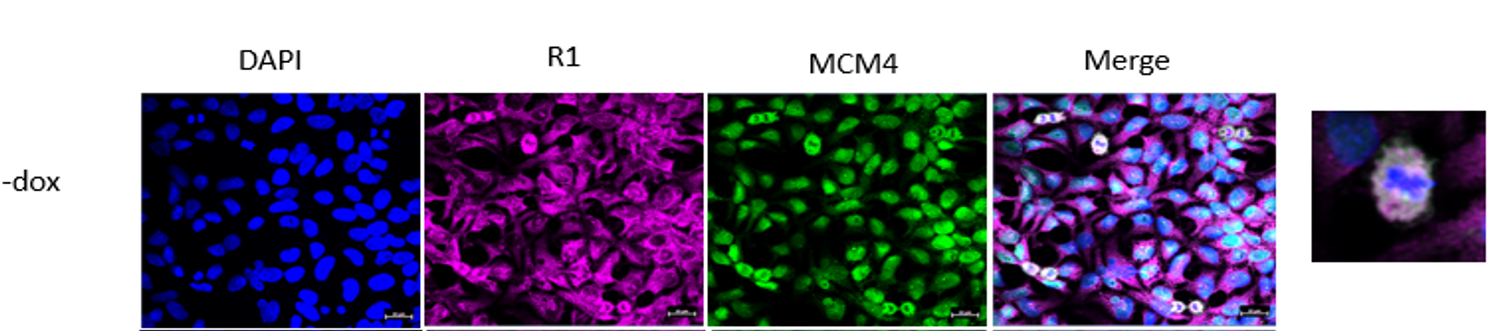
**

**
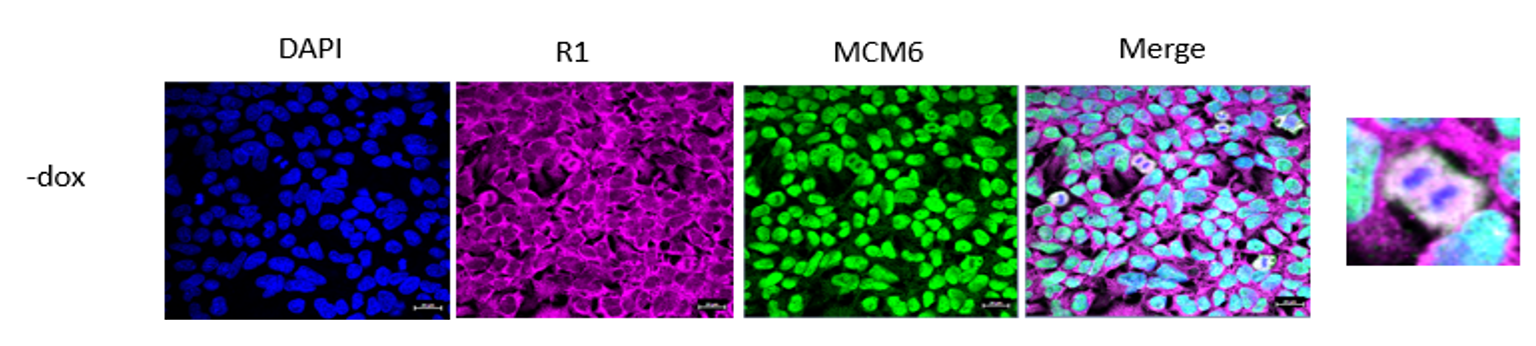
**

**
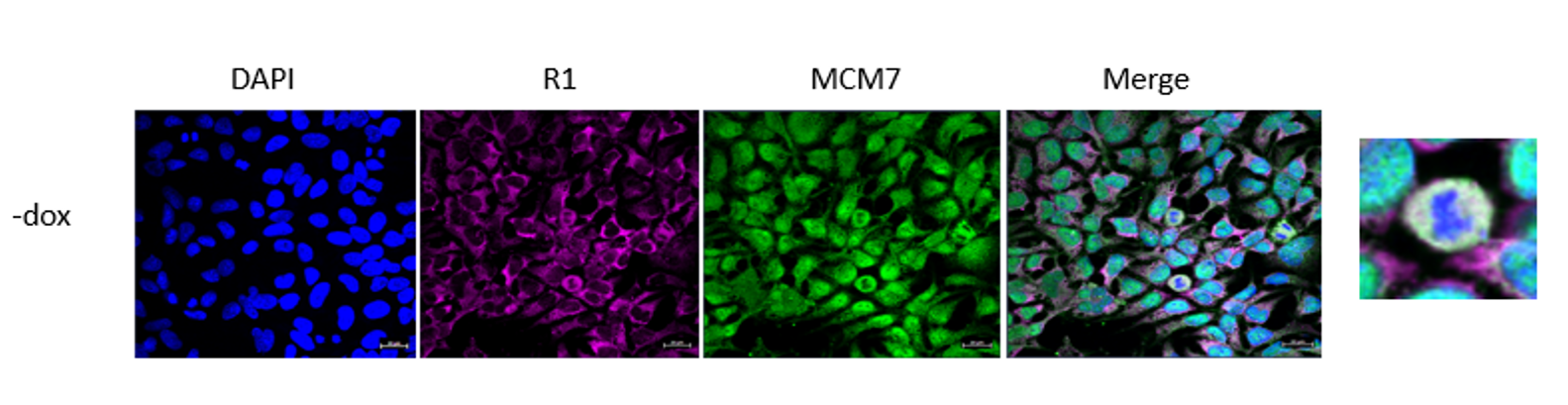
**

**Figure S8. R1 forms two complexes with MCMs: R1 and MCM7 in non-M phase cells and R1 and MCM4/6/7 in M phase (or actively dividing cells).**  High quality images of colocalization experiment from Figure 1 and a close-up (right hand side of figure) of colocalization of R1 with the individual cells in a representative cell.

IB: R1

IB: MCM7

IB: GAPDH


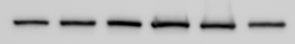

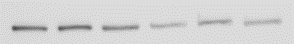

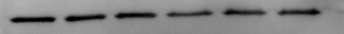

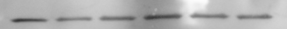

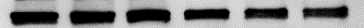

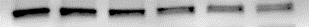


26

shR1 construct #1

Scrambled (Control)

Hours:

50

2

0


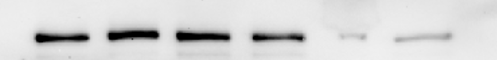

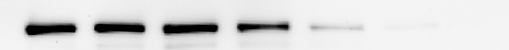

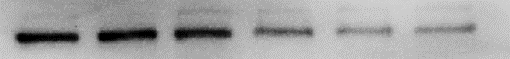

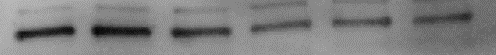


IB: MCM4

IB: MCM6

26

50

2

0

1 1.0963614 1.0629355 0.8278658

1 1.1803891 0.9635822 0.740125

1 0.9139605 0.5945327 0.4598685

1 1.0088272 0.77706735 0.3785721

1 1.0299749 0.9655087 0.8938126

1 1.0235833 0.7003811 0.5235995

**Figure S9. R1 knockdown decreases MCM7 expression.**  Representative Western blot for Figure 1E, with GAPDH-normalized densitometry values for protein expression of MCM7, MCM4, and MCM6 that were used in plotting graphs.

**References**

1 Blazquez-Bermejo, C. *et al.* Increased dNTP pools rescue mtDNA depletion in human POLG-deficient fibroblasts. *FASEB J* **33**, 7168-7179, doi:10.1096/fj.201801591R (2019).

2 Zheng, Z. *et al.* DNA synthesis and repair genes RRM1 and ERCC1 in lung cancer. *N Engl J Med* **356**, 800-808, doi:10.1056/NEJMoa065411 (2007).
